# Case of Severe Ophthalmia Cured by an Emetic

**Published:** 1814-04

**Authors:** 


*For the Medical and Physical Journal.*

### *Case of severe Ophthalmia cured by an Emetic.*

A BRITISH officer of rank had the ophthalmia whilst serving with our army in Egypt. On his return to England, he was subject to frequent and violent inflammations of his eyes, attended with much pain; for the relief of which he applied to the most eminent oculists in London, who had recourse to leeches, lancets, blisters, fomentations, and various applications—sometimes of a hot nature, and sometimes ice. This painful discipline, together with that of confinement in a dark room, he generally underwent for six or seven weeks before the inflammation was removed. Being lately in Paris, a prisoner on his parole, he was violently attacked with his old complaint, and had recourse to his old remedies; but he grew daily worse. In despair, he sent for an English surgeon that had lived for some time in that city, who, after hearing the circumstances of the case, said, "Your eyes must be left entirely to themselves; the inflammation in them I look upon as a mere secondary symptom; it is your system that must be attacked, and the chain of disease, if possible, broken." In order to which he urged his patient to take a strong emetic immediately. Against this the latter remonstrated, fearing the effect it would have upon the vessels of his eyes, already so overcharged with blood. However the surgeon was peremptory, and he submitted. The operation was violent, and the effect most happy. He got up the next morning wonderfully better. Every hour the state of his eyes improved; and in three days they were perfectly well, without any application to them but that of a little rose-water, or any additional remedy but a gentle opening medicine. Z.

